# Recombinant Human Melatonin Receptor MT1 Isolated in Mixed Detergents Shows Pharmacology Similar to That in Mammalian Cell Membranes

**DOI:** 10.1371/journal.pone.0100616

**Published:** 2014-06-24

**Authors:** Christel Logez, Sylvie Berger, Céline Legros, Jean-Louis Banères, William Cohen, Philippe Delagrange, Olivier Nosjean, Jean A. Boutin, Gilles Ferry, Frédéric Simonin, Renaud Wagner

**Affiliations:** 1 CNRS UMR7242/Laboratoire d'excellence MEDALIS, Institut de Recherche de l'ESBS, Biotechnologie et Signalisation Cellulaire, Université de Strasbourg, Illkirch, France; 2 Biotechnologie, Pharmacologie Moléculaire et Cellulaire, Institut de Recherches Servier, Croissy-sur-Seine, France; 3 CNRS UMR 5247, Institut des Biomolécules Max Mousseron (IBMM), Université de Montpellier 1 and Montpellier 2, Faculté de Pharmacie, Montpellier, France; 4 Unité de Recherches et Découvertes en Neurosciences, Institut de Recherche Servier, Croissy-sur-Seine, France; Morehouse School of Medicine, United States of America

## Abstract

The human melatonin MT1 receptor—belonging to the large family of G protein-coupled receptors (GPCRs)—plays a key role in circadian rhythm regulation and is notably involved in sleep disorders and depression. Structural and functional information at the molecular level are highly desired for fine characterization of this receptor; however, adequate techniques for isolating soluble MT1 material suitable for biochemical and biophysical studies remain lacking. Here we describe the evaluation of a panel of constructs and host systems for the production of recombinant human MT1 receptors, and the screening of different conditions for their solubilization and purification. Our findings resulted in the establishment of an original strategy using a mixture of Fos14 and CHAPS detergents to extract and purify a recombinant human MT1 from *Pichia pastoris* membranes. This procedure enabled the recovery of relatively pure, monomeric and ligand-binding active MT1 receptor in the near-milligram range. A comparative study based on extensive ligand-binding characterization highlighted a very close correlation between the pharmacological profiles of MT1 purified from yeast and the same receptor present in mammalian cell membranes. The high quality of the purified MT1 was further confirmed by its ability to activate its cognate Gαi protein partner when reconstituted in lipid discs, thus opening novel paths to investigate this receptor by biochemical and biophysical approaches.

## Introduction

The neurohormone melatonin is produced by the pineal gland at night in all mammals, whether diurnal or nocturnal [1]. With a circulating concentration in the pico-to-nanomolar range, melatonin reportedly plays a key role in controlling the circadian rhythm [2]. At much higher concentrations (micromolar and above), melatonin also modulates physio-pathological situations, such as inflammation, cancer progression, and immunological responses [3]. The actions of melatonin are mainly mediated by three binding sites [4]: MT1 and MT2, which are classical G protein-coupled receptors (GPCRs) of the class A family [5], and quinone reductase 2, which was initially described as a possible receptor (MT3) but later demonstrated to be an enzyme [6]. These sites are valuable therapeutic targets, and two melatonin MT1/MT2 agonists have recently become commercially available: Ramelteon (Takeda Pharmaceuticals, Osaka, Japan) for sleep disorder treatment [7] and Agomelatine (LLS, Suresnes, France) for depression treatment [8]. Further development of more specific and effective molecules will require biochemical and biophysical studies on purified MT1 and MT2 to achieve detailed structural and functional characterization of these receptors.

About twenty years ago, the seminal work of Brian Kobilka brought to the scientific community one purified and active recombinant member of this protein family: the beta2 adrenergic receptor [9]. This molecule was subsequently investigated using a large variety of biochemical, biophysical, and pharmacological approaches [10]–[12]. These analyses generated a wealth of data and tools, yielding major findings that have elevated our understanding of the subtle molecular mechanisms underpinning the function of this prototypical receptor. However, despite the immense interest in such approaches and the huge efforts put towards the study of other GPCRs, references and procedures describing the successful production and purification of active receptors remain rather limited. This lack of data directly relates to the fact that it continues to be highly challenging to obtain significant amounts of these membrane proteins in the purest form and retaining characteristics resembling the native proteins. Obtaining such pure preparations requires productive expression systems and efficacious extraction and purification conditions that produce homogeneous, stable, and active receptors. Moreover, few "universal" rules have been drawn from validated procedures, which are rarely transposable from one receptor to another, thereby highlighting the necessity of developing tailor-made methods for the successful production and purification of a given GPCR.

In the present paper, we describe the expression of the sequence of human MT1 in *Pichia pastoris*, as well as methods for preparing membranes, solubilization using a cocktail of detergents to maintain binding capacity, and chromatography purification to the point where MT1 appears to be the main protein species of the preparation. We further show that the purified receptor displays a pharmacological profile that closely resembles that of the membrane-bound human MT1 receptor expressed in a mammalian cell line, and that it exhibits a specific agonist-dependent G protein activation when reconstituted in lipid nanodiscs. To our knowledge, this is the first report of the purification of a functional melatonin receptor in amounts compatible with a number of protein-based analytical methodologies. Thus, this work forges a path towards improving the structural and functional characterization of MT1 at the molecular level, including the investigation of its interactions with specific ligands and protein partners.

## Experimental Procedures

### Plasmid Construction

The MT1 receptor sequence was introduced into the pDest17oi and pETG20A vectors for *Escherichia coli,* the pPIC9K vector for *Pichia pastoris*, and the pSFV2genB vector for BHK-21 cells infected with SFV as previously described [13]. To create a fusion between MT1 and the Gαi1 subunit, the MT1 receptor sequence was introduced into a vector derived from pPIC9K, in which the biotinylation domain sequence was replaced by the Gαi1 subunit sequence. To create a fusion between MT1 and YFP, the sequence Tev-YFP-His was obtained by PCR amplification and cloned into the pSFV2genB-MT1 vector in place of the sequence Tev-His. The same MT1-YFP construct was also introduced into the pcDNA5-TO vector—as was a PCR-amplified sequence of 2StrepTag-Tev-MT1—for expression in T-REx-HEK293 cells. For the cell-free/liposome expression system, the MT1 receptor sequence was introduced into two proprietary vectors from the Synthelis company, creating a His-tag either at the amino- or carboxy-terminus of the synthetized recombinant protein. For MT1 expression in the CHO-K1 cell line, the receptor cDNA was subcloned into pcDNA3.1.

### Cell Culture Procedures for MT1 Expression

For the cell-free/liposome system, the Synthelis Company produced the MT1 receptor from the two dedicated plasmids, using *E. coli* cell lysate with preformed liposomes in the presence or absence of MT1 ligands. Liposomes composed of DOPC, DOPE, cholesterol, and DMPA (Avanti Polar Lipids) in a weight ratio of 4∶2∶2∶2 were obtained by evaporation of chloroform, resuspension of the lipids, sonication, and extrusion. The reaction was performed at 30°C overnight. The generated proteoliposomes were isolated on a sucrose gradient after ultracentrifugation at 280,000 × *g* for 1 h at 4°C as previously described [14].

For MT1 expression in *E. coli*, the pDest17oi and pETG20A recombinant vectors expressing MT1 were introduced into the BL21, Rosetta, Origami, and C41 *E. coli* strains. The induction conditions for GPCR expression were as previously described [13].

For MT1 expression in *P. pastoris,* the targeted integrative transformation of a SMD1163 *P. pastoris* strain with the pPIC9K expression vectors, the selection of recombinant yeast clones, and the standard culturing procedures were performed as previously described [15]. Methanol-induced MT1 expression was carried out at an initial OD_600_ of 5 in BMMY medium supplemented with 1 *µ*M D600, 3% DMSO, and 0.4 mg/mL histidine. After 18 h induction at 20°C, cells were harvested at 3,000 × *g* for 10 min, washed with phosphate-buffered saline (PBS; 20 mM sodium phosphate pH 7.4, 2 mM KCl, and 150 mM NaCl) and subsequently used for membrane preparation.

To culture SFV-infected BHK-21 cells, recombinant SFV particles were generated and BHK-21 cells in suspension culture were infected as previously described [16]. At 48 h post-infection, cells were pelleted at 1,000× *g* for 10 min, washed with PBS, and subsequently used for membrane preparation.

The inducible stable T-REx-HEK293 cell lines expressing MT1 were established as previously described [17]. Cells were grown in flasks at 37°C under a humidified 5% CO_2_ atmosphere in DMEM/F-12 medium supplemented with 10% FBS, 4 mM Glutamax, 10 *µ*g/mL blasticidin, and 200 *µ*g/mL hygromycin. When cells reached 90–100% confluence, receptor expression was induced by the addition of 2 *µ*g/mL tetracycline and 5 mM of sodium butyrate. At 48 h after induction, cells were detached with 5 mM EDTA in PBS, pelleted by centrifugation at 1,000× *g* for 10 min, washed with PBS, and subsequently used for membrane preparation.

The CHO-K1 cell line stably expressing the human MT1 receptor [18] was grown to confluence and harvested in PBS containing 5 mM EDTA. After centrifugation at 1,000× *g* for 20 min at 4°C, the resulting pellet was subsequently used for membrane preparation.

### Membrane Preparation

For *P. pastoris*, all procedures were performed on ice. The yeast cells were resuspended in cold lysis buffer (50 mM Tris pH 7.4, 0.5 M NaCl, 10% glycerol, 1 mM EDTA, and 1 mM PMSF). Cells were then lysed with three cycles of 60-s shaking and 60-s cooling on ice, using 0.5-mm glass beads in a FastPrep 24 device. Unbroken cells and cell debris were removed by centrifugation at 3,000 × *g* for 10 min, and the supernatant containing the membrane fraction was pelleted by ultracentrifugation at 100,000 × *g* for 45 min at 4°C. Membrane pellets were resuspended in a cold membrane buffer (50 mM Tris pH 7.4, 0.5 M NaCl, 10% glycerol, and 1 mM PMSF) using a Dounce homogenizer, and stored at −80°C.

For HEK293 and BHK-21 cells, all procedures were again performed on ice. The cells were resuspended in a cold lysis buffer (50 mM Tris pH 7.4, 0.5 M NaCl, 10% glycerol, 2 mM MgCl_2_, 1 mM EDTA, and 1 mM PMSF), and the cells were then lysed using an Ultra-Turrax T25 homogenizer. Membranes were pelleted by ultracentrifugation at 100,000 × *g* for 45 min at 4°C. Membrane pellets were homogenized in cold membrane buffer (50 mM Tris pH 7.4, 0.5 M NaCl, 10% glycerol, 2 mM MgCl_2_, and 1 mM PMSF) with Ultra-Turrax T25 and then ultracentrifuged once again. Finally, the membranes were resuspended in cold membrane buffer using a Dounce homogenizer, and stored at −80°C.

CHO cells were resuspended in 5 mM Tris-HCl (pH 7.4) containing 2 mM EDTA, and were homogenized using a Kinematica polytron. The homogenate was then centrifuged at 20,000 × *g* for 30 min at 4°C, and the resulting pellet was resuspended in 75 mM Tris-HCl (pH 7.4) containing 2 mM EDTA and 12.5 mM MgCl_2_. Aliquots of membrane preparations were stored at −80°C until use.

### Purification of the MT1 Receptor Expressed in *P. pastoris*

For analytical-scale purification, membrane proteins were diluted to 2 mg/mL in cold solubilization buffer (50 mM HEPES pH 7.4, 0.5 M NaCl, 1 mM PMSF, and 1 *µ*M D600) supplemented with different concentrations of a detergent/cholesteryl hemisuccinate (CHS) mixture (1/0.1 w/w). The suspension was incubated for 5 min at room temperature, followed by centrifugation at 100,000 × *g* for 45 min to pellet the non-solubilized material. Solubilized proteins were then purified on Ni-NTA spin column following the manufacturer's protocol. The column was briefly equilibrated in a purification buffer (50 mM HEPES pH 7.4; 0.5 M NaCl; 1 mM PMSF; 1 *µ*M D600; and 0.1% detergent/0.01% CHS for DM, DDM, and Fos14 or 0.5% detergent/0.05% CHS for CHAPS) supplemented with 20 mM imidazole. Solubilized proteins were loaded onto the column, washed with purification buffer supplemented with 20 mM imidazole, and finally eluted in a final volume of 100 *µ*L elution buffer (purification buffer supplemented with 300 mM imidazole). Next, 10 *µ*L of purified proteins were loaded onto an analytical Superdex 200 5/150 GL (GE Healthcare) pre-equilibrated with purification buffer on an ÄKTA FPLC system. Proteins were separated in the purification buffer at 0.3 mL/mn, and the absorbance was measured at 280 nm.

For preparative-scale purification, membrane proteins were also diluted and solubilized following the above-described procedure. Solubilized proteins were then incubated in batches with 1 mL of anti-FLAG M2 affinity gel prepared following the manufacturer's instruction. The suspension was next transferred to a 10-mL drip gravity-flow column (Bio-Rad), the flow-through was collected, and the resin was washed with 25 mL of purification buffer (50 mM HEPES pH 7.4, 0.5 M NaCl, and 1 *µ*M D600 supplemented with the detergent/CHS mixtures as described above). Bound proteins were eluted in the same buffer containing 100 *µ*g/mL of FLAG peptide. The pooled anti-FLAG affinity chromatography elution fractions were concentrated down to 200–250 *µ*L in a centrifugal concentrator with a 50-kDa MWCO. The concentrated sample was then loaded onto a Superdex 200 10/300 GL pre-equilibrated with the same purification buffer on an ÄKTA FPLC system. Proteins were separated in the purification buffer at 0.3 mL/mn, and 0.5-mL fractions were collected.

### Protein Dosage, SDS-PAGE, and Western Blot

Protein concentrations were determined using the bicinchoninic acid assay with bovine serum albumin as a standard. Protein samples were diluted in NuPAGE LDS sample buffer, separated on a NuPAGE 10% Bis-Tris gel with MOPS buffer, and directly stained with Coomassie blue or electrotransferred to a nitrocellulose membrane for 1 h at 100 V. After blocking with BLOT-Quick Blocker (GE Healthcare), the nitrocellulose membrane was incubated with a M2 anti-FLAG antibody (diluted 1∶8,000) and revealed with an HRP-conjugated anti-mouse secondary antibody (diluted 1∶10,000). Finally, the immunoblots were visualized using an enhanced chemiluminescence reagent following the manufacturer's procedure.

### Electron Microscopy

The protein samples were diluted to approximately 50 *µ*g/mL in purification buffer. Negative staining was performed using 2% (w/v) silicotungstate sodium (pH 7.4) with the floating mica technique. Observation was carried out using a transmission electron microscope Philips CM 120 with a filament LaB6 (lanthanum hexaboride) at 120 kV. Images were recorded at 45,000× magnification using a Gatan Orius CCD camera.

### Ligand Binding

Ligand binding experiments were carried out as previously described [19]. Briefly, the membrane protein samples (50 *µ*g/mL) were incubated in 96-well plates for 2 h at 25°C in binding buffer (50 mM Tris-HCl pH 7.4, 5 mM MgCl_2_, and 1 mM EDTA). For saturation assays, a concentration range of 0.025 to 50 nM [^3^H]-melatonin was used as the tracer, and non-specific binding was determined with 10 *µ*M melatonin. In competition experiments, the [^3^H]-melatonin concentration was maintained at 5 nM, and competitor molecules were assayed in the range of 10^−15^ to 10^−3^ M. After incubation, the reaction was stopped by rapid filtration through GF/B unifilters, followed by three successive washes with ice-cold 50 mM Tris-HCl (pH 7.4).

For purified proteins, the same protocol was followed with the following modifications: the concentration of purified proteins was 0.1 *µ*g/mL, a solution of 0.1% CHAPS/0.01% CHS was added to the binding buffer, and 1 nM [^3^H]-melatonin was applied for competitive assays. Additionally, proteins were precipitated after incubation by supplementation with 0.1% gamma globulins and 25% PEG 6000 (Sigma) for 15 min before filtration on GF/B filters. Then the proteins were washed three times with ice-cold buffer containing 50 mM Tris-HCl (pH 7.4) and 8% PEG 6000. Data were analyzed with GraphPad PRISM Software. For the saturation assay, binding site density (Bmax) and the dissociation constant of the radioligand (K_D_) were calculated according to the method of Scatchard. For competition experiments, inhibition constants (Ki) were calculated according to the Cheng-Prussof equation: Ki = IC50/[1 + (L/K_D_)], where IC50 is the inhibitory concentration 50% and L is the concentration of [^3^H]-melatonin. The Ki values were expressed as pKi, corresponding to the logarithmic expression of Ki [pKi = −log(Ki)], and the Pearson product-moment correlation coefficient was employed for statistical correlation analysis of pK_i_ values.

### Nanodisc assembly

The membrane scaffold protein MSP1E3D1(-) was purified as previously described [20]. MSP1E3(-) was mixed at a 1∶90 molar ratio with purified lipids (POPC/POPG; 3/2 molar ratio) previously dissolved at a 24 mM concentration in a 20 mM HEPES, 100 mM NaCl, 1 mM EDTA, 48 mM Na-cholate, pH 7.5 buffer. The mixture was incubated for 15 minutes on ice. The purified receptor was then added to the MSP:lipid mixture at 0.1∶1 receptor:MSP1E3(-) molar ratio and further incubated for 60 minutes on ice. Self-assembly was initiated by detergent removal using BioBeads SM-2 (Biorad) (0.5 g of Biobeads per mL of reconstitution mixture) and allowed to proceed for four additional hours. The Biobeads were then removed by centrifugation and the recovered supernatant was directly loaded on a 1 mL HisTrap column (GE Healthcare) previously equilibrated in a 25 mM Tris-HCl, 300 mM NaCl, 10 mM imidazole, pH 8 buffer. After extensive washing with the equilibration buffer, the MT1R-containing discs were eluted with the same buffer containing 250 mM imidazole. The discs were finally purified using size-exclusion chromatography. To this end, the fractions recovered from the HisTrap column were concentrated and loaded on a Superdex 200 10/300 GL column (GE Healthcare) previously equilibrated in a 25 mM HEPES, 200 mM NaCl, pH 7.5 buffer. Fractions eluted from the column (flow rate of 0.2 mL/min) were pooled and directly used in the Gi activation assays.

### *In vitro* Gi activation assays

The nucleotide-exchange assay using the purified Gαi subunit was carried out as described by Hamm and colleagues [21]. Gαi and the β1γ2 subunits of the G protein were prepared as described [22], [23]. The basal rate of GTPγS binding was determined by monitoring the relative increase in the intrinsic fluorescence (λexc = 300 nm, λem = 345 nm) of Gαi (500 nM of purified Gαi2) in the presence of purified Gβ1γ2 subunits (500 nM) and of empty discs (100 nM) in a buffer 25 mM Hepes, 200 mM NaCl, 2 mM MgCl2, pH 7.5 for 30 min (1 min steps) at 15°C after the addition of 10 µM GTPγS. We checked that the MT1R agonist alone did not affect the basal rate of Gαi activation by carrying the same experiment in the presence of 10 *µ*M melatonin. The receptor-catalyzed rate was measured under the same conditions using MT1R-containing discs (100 nM) in the absence or in the presence of 10 *µ*M melatonin.

## Results

### Evaluation of a Panel of MT1 Recombinant Constructs and Sources of Production

Performing *in vitro* studies of proteins requires significant amounts of material, typically in the mg range. In the case of membrane proteins and GPCRs in particular, this systematically implies the use of recombinant systems that efficiently overexpress the gene of interest. Since no universal system is readily available for this purpose, the first required task is to identify the best recombinant sequence to introduce into the most appropriate production system. To successfully produce the human melatonin MT1 receptor, we selected a panel of five representative expression systems that were proven to be efficient for overexpressing several GPCRs. These included a bacterial cell-free technology in which the expressed receptors are directly embedded in liposomes [24], the popular *E. coli* bacterial system [13], the eukaryotic microorganism *Pichia pastoris* [15], and two mammalian cell lines: one used for transient expression (BHK cells infected by a recombinant SFV alphavirus [16]) and another for stable and inducible expression (T-REx system with HEK293 cells [17]). We additionally fused various tag sequences to the receptor cDNA based on their proven benefits relating to detection, purification, and/or receptor stability.

**Figure 1:**
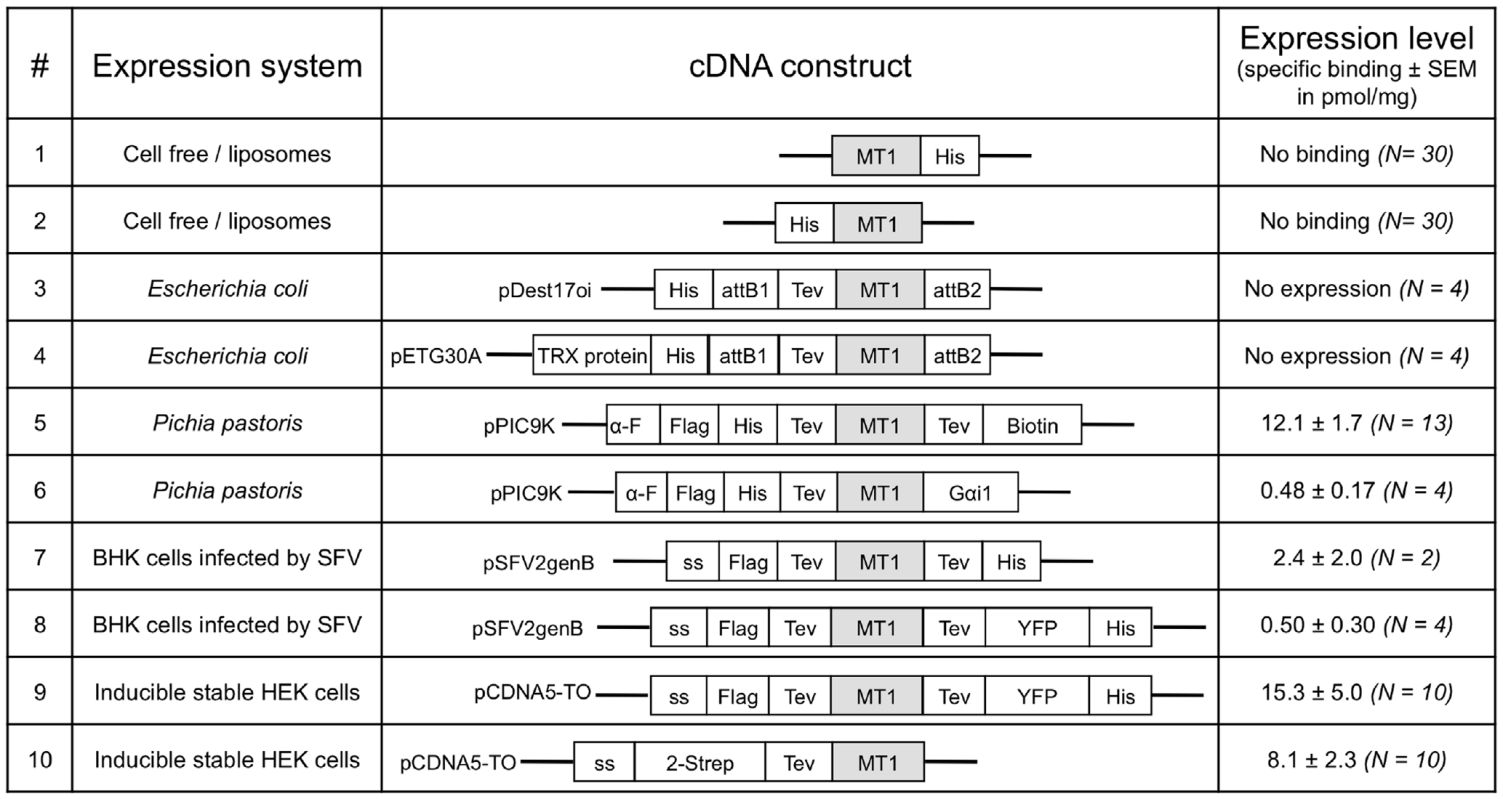
MT1 expression yields obtained with ten combinations of cDNA constructs and host systems. Expression levels of the human MT1 receptor were assessed using a [^3^H]-melatonin ligand binding assay. N: number of independent experiments. Schematic representations of the evaluated expression vectors use the following abbreviations: MT1, human MT1 receptor; His, 10-histidine tag; attB1 and attB2, recombination sites of the Gateway system; Tev, tobacco etch virus protease cleavage site; Trx protein, thioredoxin protein; α-F, sequence signal of the *Saccharomyces cerevisiae* α-Factor; Flag, flag-epitope tag; Biotin, biotinylation domain from *Propionibacterium shermanii*; Gαi1, αi1 subunit of G protein; ss, signal sequence from influenza hemagglutinin gene; YFP, yellow fluorescent protein; and 2-Strep, double Strep tag.

Figure 1 summarizes the ten combinations of constructs and host systems that were evaluated in this study, and for which we assessed the number of MT1 receptor binding sites using a specific radioactive ligand binding assay. The results clearly indicate poor performance of prokaryotic systems, since no specific ligand binding was measured for samples obtained with the bacterial cell-free technology or with *E. coli* extracts, with which receptor polypeptide was not even immunodetected (data not shown). The output was much more promising from the eukaryotic systems with specific [^3^H]-melatonin binding detected in all tested conditions. Receptors transiently expressed in SFV-infected BHK cells (Fig. 1, #7 and 8) displayed more fluctuating expression levels than in the yeast and HEK eukaryotic systems (Fig. 1, #5, 6, 9, and 10). Membranes from inducible stable HEK cells (Fig. 1, #9) and from *P. pastoris* (Fig. 1, #5) displayed the highest levels of ligand binding, with more than 15 and 12 pmol/mg of proteins, respectively. These results also highlighted the impact of the selected fusion sequences on the expression output for a given host cell—for instance, showing a 25-fold variation of ligand binding receptors between the two constructs expressed in *P. pastoris* (Fig. 1, #5 and 6).

Overall, this limited but representative screening of expression systems allowed us to identify inducible stable HEK cells (Fig. 1, #9) and *P. pastoris* (Fig. 1, #5) as the best production systems for obtaining ligand-binding active MT1 receptor. However, in our hands, the former cell system couldn't be adapted to a cell suspension culturing format suitable for large-scale production needs. Conversely, the yeast *Pichia pastoris* was fully compatible with mass production approaches, and thus appeared to be the most appropriate for the generation and study of the purified MT1 receptor. In typical experiments, from 1 L of culture, we obtained about 500 mg of total membrane proteins, containing an average of 6,500 (±850) pmol of ligand binding receptors.

### Screening Conditions for Extraction of MT1 from *Pichia pastoris* Membranes

**Figure 2:**
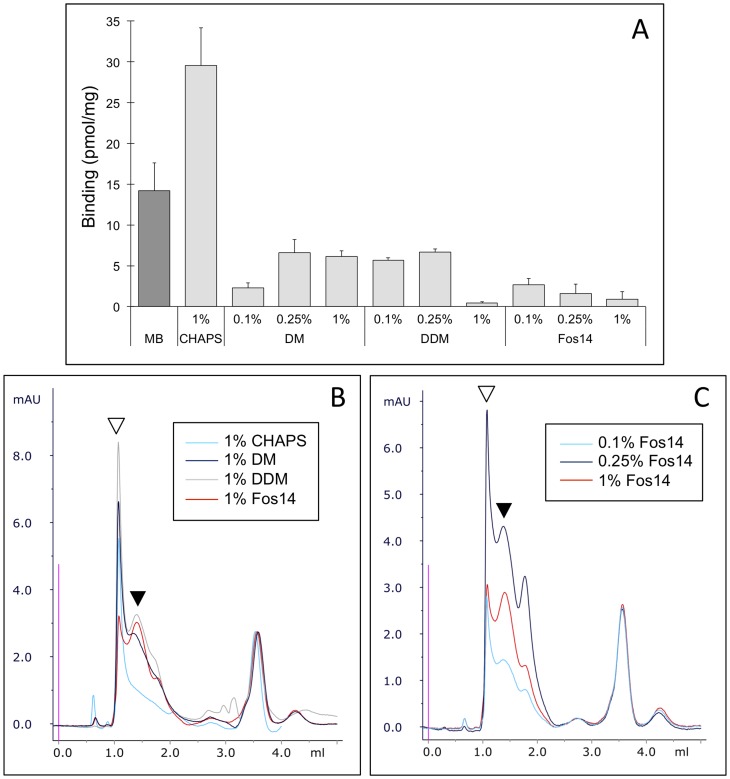
Detergent screening for MT1 extraction from *Pichia pastoris* membranes. *P. Pastoris* membranes were solubilized in the presence of a panel of detergent concentrations indicated on the figures. Solubilized proteins were then partially purified on Ni-NTA Spin columns, and finally analyzed using a [^3^H]-melatonin binding assay (*A*) and analytical size exclusion chromatography (SEC) (*B* and *C*). ***A***, MB: *P. pastoris* membranes expressing MT1 receptor. ***B*** and ***C***, Protein absorbance profiles measured at 280 nm; white triangles: MT1 oligomers; black triangles: MT1 monomers.

As a first step towards purification, we briefly screened a variety of detergents to identify the best conditions for efficient extraction of active and homogeneous MT1. Based on our previous experience with GPCR solubilization from *P. pastoris* membranes, this screening focused on a limited number of conditions, including four representative detergents—CHAPS, DM, DDM, and Fos14—which we tested at different concentrations (Fig. 2). Each solubilization condition was tested with the same amount of starting membrane sample (MB), and the resulting samples were analyzed for MT1 receptor activity (ligand binding assay, Fig. 2A) and homogeneity (analytical SEC run after IMAC purification on spin columns, Fig. 2B). These experiments highlighted three typical detergent behaviors. CHAPS allowed extraction of the highest amounts of active MT1, but mainly in an oligomeric state according to the SEC calibration data (Fig. 2B, white triangle). At the other extreme, Fos14 enabled recovery of the highest ratio of monomeric receptors (Fig. 2B, black triangle), but with very low ligand binding activity. The other two detergents—DM and DDM—resulted in intermediate situations. Additionally, we found that both the activity and the polydispersity of the receptor were diversely impacted by the detergent concentration tested—except for CHAPS for which only one concentration could be assayed due to its high critical micelle concentration (CMC; close to 0.5%). For example, increasing concentrations of Fos14 enabled the recovery of higher proportions of monomeric MT1 as assessed by SEC analysis (Fig. 2C). We further found that high concentrations of Fos14 or DDM, which both have a very low CMC, were severely detrimental to the ligand binding activity (Fig. 2A). Overall, our solubilization screening resulted in the identification of a panel of representative situations for MT1 extraction that could be further investigated for purification purposes.

### Purification of a Ligand-Binding Active and Monomeric MT1 Receptor

The detergent screening was followed by preliminary purification attempts. As DM and DDM showed a good compromise between ligand binding activity and significant amounts of monomeric receptors after membrane extraction, these detergents were first utilized in affinity purification approaches. However, the output was disappointing in all cases, achieving only low yields of <50% pure receptor with very poor ligand binding activity (data not shown).

**Figure 3:**
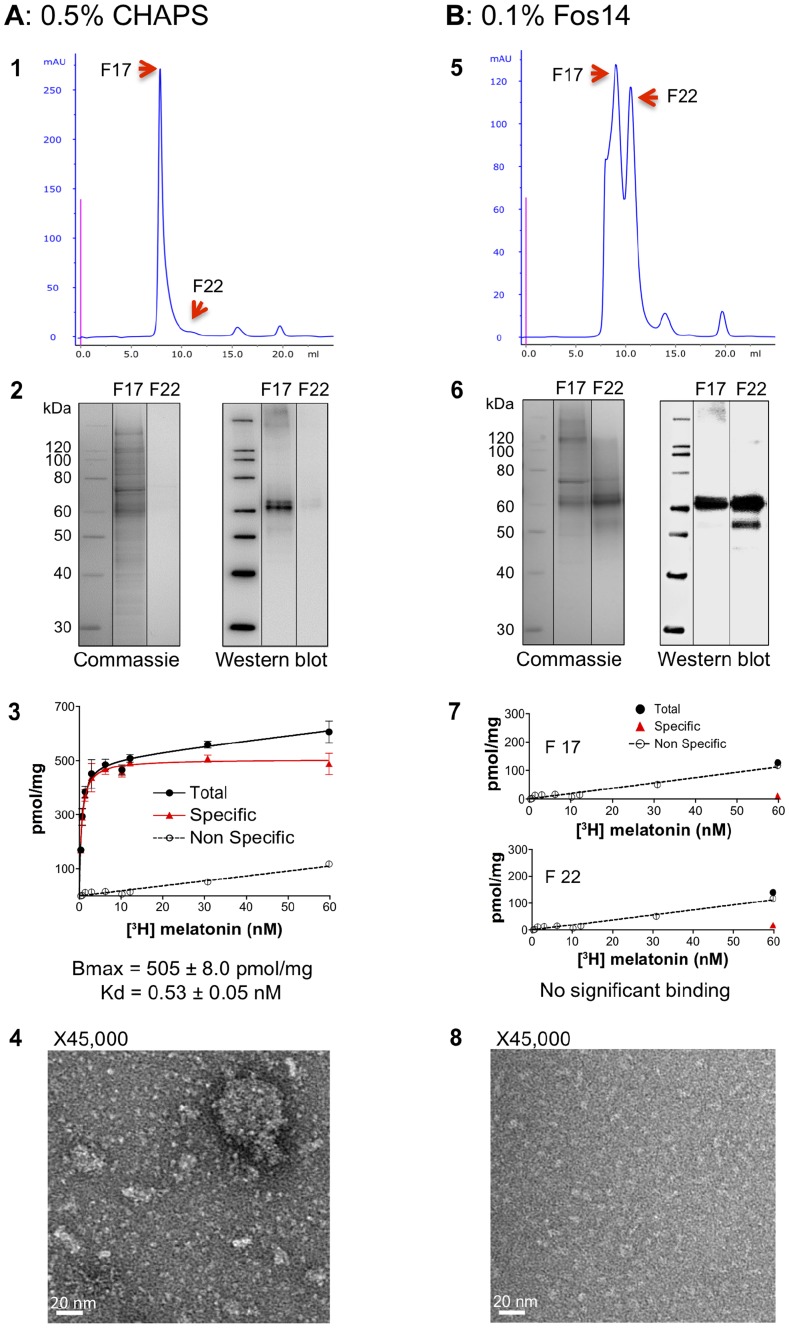
Characterization of MT1 samples purified in the presence of CHAPS or Fos14. *P. pastoris* membranes were solubilized with 1% CHAPS (***A***) or 0.25% Fos14 (***B***), and purified in the presence of the indicated concentration of detergents using a two-step purification approach consisting of anti-flag affinity chromatography followed by size exclusion chromatography. **1 and 5**: Size exclusion chromatography profile. Red arrows indicate SEC elution fractions F17, corresponding to MT1 oligomers, and F22, corresponding to MT1 monomers. **2 and 6**: SDS-PAGE of SEC elution fractions F17 and F22 colored with Coomassie Blue (left) and revealed by anti-Flag immunodetection (right). **3 and 7**: Saturation ligand binding experiments with [^3^H]-melatonin on SEC elution fraction F17 for CHAPS and F17 or F22 for Fos14. **4 and 8**: Negative staining electron microscopy on SEC elution fractions F17 for CHAPS and F22 for Fos14.

Therefore, we next focused on MT1 receptor samples solubilized with the two other detergents: CHAPS and Fos14. In parallel experiments, each was used during solubilization and maintained throughout a two-step purification approach consisting of anti-flag affinity chromatography, followed by size exclusion chromatography. Figure 3 presents the results. Consistent with our findings in the solubilization screening, CHAPS mainly allowed the recovery of high molecular weight particles containing partially purified MT1, as assessed by SEC and SDS-PAGE analyses (Fig. 3A, panels 1 and 2; Figure S1). However, the obtained saturation curve revealed significant ligand binding activity of the oligomeric MT1 contained in the F17 fraction of the SEC column (Fig. 3A panel 3). Conversely, Fos14 enabled retrieval of a significant monomeric receptor population (fraction F22 on Fig. 3B, panel 5) with a higher level of purity (Fig. 3B, panel 6; Figure S2), but with complete loss of ligand binding activity (Fig. 3B, panel 7). Electron microscopy (EM) analysis further confirmed the presence of a number of objects of various sizes—including aggregates and probably small remains of membranes—in samples obtained in the presence of CHAPS, while those from purification with Fos14 appeared much more homogeneous (Fig. 3, panels 4 and 8, respectively). Altogether, these results suggested that Fos14 was well suited for extracting individual but probably denatured receptors, while CHAPS likely extracted complex protein samples that provided a more suitable environment for maintaining MT1 activity.

**Figure 4:**
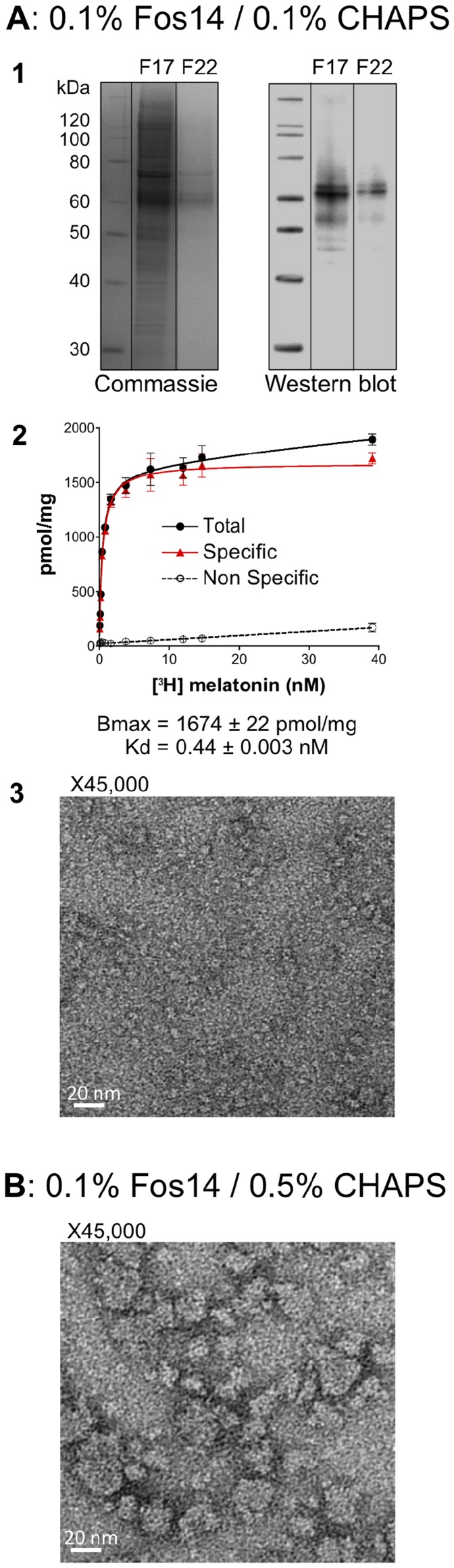
Characterization of samples purified in a mixture of CHAPS and Fos14 detergents. *P. pastoris* membranes were solubilized and purified using a two-step purification approach (anti-flag affinity chromatography followed by SEC). ***A***, Samples were solubilized in a mixture of 0.25% Fos14/0.1% CHAPS and purified in presence of 0.1% Fos14/0.1% CHAPS. **1**: SDS-PAGE of SEC elution fractions F17 and F22 colored with Coomassie Blue (left) and revealed by anti-Flag immunodetection (right). **2**: Saturation ligand binding experiments with [^3^H]-melatonin on SEC elution fraction F22. **3**: Negative staining electron microscopy on SEC elution fraction F22. ***B***, Samples were solubilized in a mixture of 0.25% Fos14/1% CHAPS, purified in the presence of 0.1% Fos14/0.5% CHAPS, and analyzed by negative staining electron microscopy.

These findings prompted us to design a mixed-detergent extraction and purification strategy involving both Fos14 and CHAPS, with the aim of retaining only the beneficial properties of each detergent. We thus screened the use of various Fos14-to-CHAPS ratios during solubilization and purification, and analyzed each sample in ligand binding experiments (saturation curves) as well as by SEC, SDS-PAGE, and EM. Notably, we found that a mixture of 0.25% Fos14 and 0.1% CHAPS during the solubilization step, followed by 0.1% Fos14 and 0.1% CHAPS during IMAC and SEC purification allowed the isolation of relatively pure, monomeric, and active MT1 receptor (Fig. 4A; Figure S3). In typical experiments, we routinely recovered about 150 to 200 *µ*g (BCA assay quantification) of monomeric MT1 from 100 mg of total membrane protein preparations, which represents about 0.8 to 1 mg of purified receptor starting from 1 liter of cultured yeast. Moreover, the Fos14/CHAPS combination led to the retrieval of higher amounts of ligand-binding MT1 compared with receptors obtained with CHAPS alone (1674±22 *versus* 505±8 pmol/mg) and the recovered samples were also much more homogeneous, with EM images showing no apparent presence of aggregated particles (Fig. 4A, panel 3 *versus* Fig. 3A, panel 4). Interestingly, samples obtained with higher concentrations of CHAPS in the detergent mixture during solubilization and purification exhibited rather homogeneous populations of oligomeric or aggregated forms of the receptor (Fig. 4B). Overall, these data clearly indicated that a mixture of CHAPS and Fos14 detergents during solubilization and purification of MT1 would substantially increase the yield and quality of purified receptor samples, as compared to the use of these detergents separately.

### Pharmacological Analysis of the MT1 Receptor Purified in Mixed Detergent Micelles

**Figure 5:**
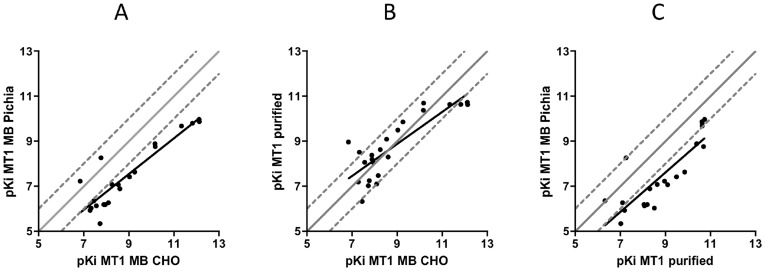
Correlation plots between binding affinities of MT1 receptors in CHO membranes, in *P. pastoris* membranes, and purified. ***A***, pKi correlation of MT1 in *P. pastoris* membranes *vs.* MT1 in CHO membranes. ***B***, pKi correlation of MT1 purified in Fos14/CHAPS *vs.* MT1 in CHO membranes. *C*, pKi correlation of MT1 in *P. pastoris* membranes *vs.* MT1 purified in Fos14/CHAPS. Processed data are presented in Table 1. Pearson's correlation analyses revealed r coefficients of 0.908 (*p*<0.0001, n = 4), 0.840 (*p*<0.0001, n = 4), and 0.840 (*p*<0.0001, n = 4) for *A*, *B*, and *C*, respectively.

In the next series of experiments, we analyzed the pharmacology of the MT1 purified with mixed detergents. Using a [^3^H]-melatonin ligand binding assay, we measured the affinity of a set of 24 compounds representative of melatonin receptor agonists and antagonists for which affinities were already published for human, sheep, rat, and mouse melatonin receptors [18], [25]–[28]. Competition experiments were performed to assay membranes from CHO cells (n > 3) and *P. pastoris* (n = 3) expressing the human MT1 receptor, as well as four independent samples from *P. pastoris* membranes purified with a 0.1% Fos14/0.1% CHAPS mixture, with MT1 from CHO membranes serving as a reference (Table 1). Dose-response curves were analyzed by a non-linear regression from which pKi values were determined (Table 1). These pKi values were further compared via Pearson correlation analyses between MT1 in CHO and *P. pastoris* membranes, between MT1 in CHO membranes and purified MT1, and between MT1 in *P. pastoris* membranes and purified MT1 (Fig. 5, panels A, B and C, respectively). Our results demonstrated markedly decreased ligand binding affinities of both agonists and antagonists for MT1 in *P. pastoris* membranes compared to MT1 in CHO membranes. However, correlation analysis showed that the affinity ranking toward these compounds was fully maintained (r = 0.908). Most strikingly, the receptor purified from the yeast membranes in a Fos14/CHAPS mixture revealed a pharmacological profile much closer to that of the receptor in CHO membranes (Fig. 5, panel B) than in yeast membranes (Fig. 5, panel C). These results support that using the chosen detergent mixture during the solubilization and purification process is an efficient strategy for recovering a purified human MT1 receptor with ligand binding characteristics close to the native form, even if the starting *P. pastoris* membranes display receptors with lower affinities.

**Table 1:** Comparison of binding affinities (Ki) of MT1 receptors in CHO and *Pichia pastoris (P.p.)* membranes, and purified in a 0.1% Fos14/0.1% CHAPS mixture.

	MT1 CHO membranes	MT1 *P. p*. membranes	MT1 purified
	pKi ± SEM	pKi ± SEM	pKi ± SEM
MLT	10.15±0.12	8.88±0.02	10.38±0.15
2I-MLT	12.12±0.20	9.87±0.01	10.62±0.06
4P-PDOT	7.56±0.16	6.13±0.01	8.06±0.21
Luzindole	8.09±0.31	6.27±0.01	7.10±0.12
FLN68	11.82±0.06	9.80±0.01	10.63±0.12
SD6	11.33±0.34	9.68±0.01	10.63±0.11
6-Cl-MLT	9.25±0.07	7.63±0.01	9.85±0.17
2-Br-MLT	12.11±0.08	9.97±0.01	10.73±0.16
S 70254	7.32±0.31	6.03±0.01	8.51±0.12
SD1881	6.83±0.24	7.23±0.13	8.96±0.15
SD1882	7.95±0.07	6.19±0.01	8.04±0.12
SD1918	7.88±0.10	6.19±0.17	8.19±0.09
S 22153	8.25±0.09	7.08±0.14	8.63±0.15
S 27128	9.03±0.12	7.42±0.15	9.49±0.19
S 20098	10.17±0.25	8.76±0.10	10.69±0.17
D600	7.76±0.15	8.26±0.09	7.25±0.06
DIV880	7.44±0.12	6.35±0.01	6.32±0.05
5HT	<5	<5	<5
S 20928	7.27±0.26	5.93±0.04	7.19±0.13
S 75436	8.53±0.06	7.07±0.01	9.09±0.21
S 21278	7.71±0.14	5.34±0.01	7.02±0.14
S 73893	8.60±0.06	6.89±0.01	8.30±0.13
S 77834	7.87±0.15	nd	8.38±0.14
S 77840	8.16±0.11	nd	7.47±0.19

Twenty-four compounds from our MT1 ligands collection were tested. 4P-PDOT, Luzindole, S 22153, D600, S 73893-1, S 77834, and S 77840 are antagonist ligands, while the others are agonist ligands. Concentration-response curves were analyzed by non-linear regression. Binding affinities are expressed as mean pKi ± SEM of four independent experiments.

### The Purified MT1 Receptor Reconstituted in Nanodiscs Activates G Proteins

**Figure 6:**
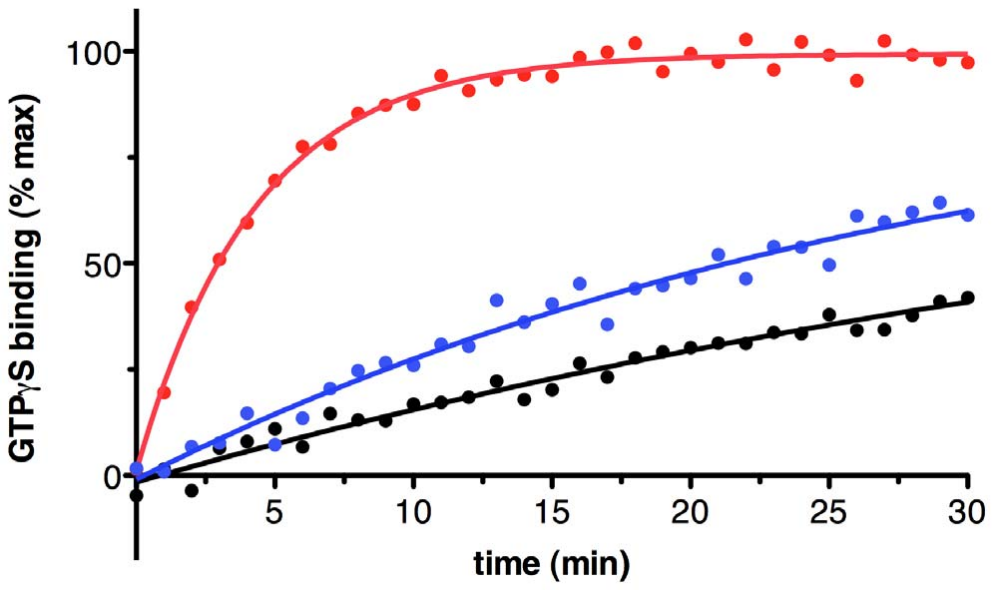
Gi coupling of MT1 reconstituted in lipid nanodiscs. Kinetics of GTPγS binding to Gi in the Gαiβ1γ2 trimer triggered by MT1R-containing nanodiscs in the absence of ligand (open circles) or in the presence of 10 µM of the melatonin agonist (closed circles). The kinetics measured in the presence of empty nanodiscs is given as open squares. These data were treated as described in the Materials and Methods section to extract the basal exchange rates.

The MT1 receptor purified with the Fos14/CHAPS mixture was further used for reconstitution in lipid discs as previously described for other GPCRs such as rhodopsin [20], mGluR2 [29] or GHS-R1a [30]. To assess the functionality of the purified receptor after assembly into lipid discs, we measured receptor-catalyzed GTPγS binding to Gαi using the assay developed by Hamm and colleagues [21]. As shown in Fig. 6, a significant increase in GTPγS binding was observed when the MT1-containing nanodiscs were assayed in presence of melatonin, in comparison with the basal signal measured in the absence of receptor. The GTPγS exchange rate value measured under such conditions is of 0.23±0.02 min^−1^, to be compared to the value inferred for the isolated G protein in the presence of empty discs (0.02±0.01 min^−1^). Of importance, this value is consistent with what has been reported for other isolated receptors [20], [29], indicating that the MT1R into the lipid discs is likely to be stabilized in a native fold. This is further confirmed by the observation of a slight but significant constitutive activation of Gαi in the presence of the ligand-free receptor. Indeed, this is consistent with the constitutive activity that has been reported for the human MT1 receptor studied in a cellular environment [31].

## Discussion

Although the last decade has seen important efforts made towards the production of recombinant GPCRs for *in vitro* studies, identifying experimental conditions for obtaining pure and active receptors is still handled case by case and using trial and error [32]. Accordingly, the present study required successive screenings applied at each step of the production procedure to determine how to recover near-milligram amounts of a relatively pure and ligand-binding competent human MT1 melatonin receptor. The resulting tailor-made MT1 production process initially relies on the use of the *P. pastoris* yeast system, which has been repeatedly proven to be particularly well-adapted to the overexpression of a large panel of eukaryotic membrane proteins, including GPCRs, at a crystallization-grade level [33]–[35]. As emphasized in our study, this very flexible system not only combines advantageous handling and upscaling properties, but also offers cellular machineries and a membrane environment that are comparably efficient to a HEK mammalian cell host for high-level MT1 expression.

In addition to using this effective bioproduction system, our MT1 production procedure implements an original method of combining the Fos14 and CHAPS detergents for optimally extracting and maintaining the receptor in a soluble and ligand-active state. Although DDM has been widely used for GPCR structural studies in all recent reports, the two maltoside-derived detergents that we tested enabled the recovery of only low amounts of partially purified MT1. Thus, in the present study, we investigated and exploited the opposite and complementary properties of Fos14 and CHAPS on MT1 extraction and solubility. We found that Fos14 was the most potent detergent for extraction and maintenance of MT1 in its monomeric form, albeit with poor ligand binding activity. On the other hand, CHAPS was best suited for retaining the activity of soluble MT1 receptors but in a more polydisperse and heterogeneous shape. These two zwitterionic detergents are not frequently employed for membrane protein extraction and purification, but comparable behaviors have been previously reported. One study evaluated 110 detergents for solubilization of the protective antigen (a heptameric pore-forming membrane protein), and reported that Fos14 was the only detergent to enable solubilization and maintenance in a monodisperse form [36]. A similar detergent screening approach demonstrated Fos14 and Fos16 to be the most effective surfactants for the solubilization and the purification of the tetrameric human multidrug transporter ABCG2, even though the activity of the soluble protein appeared rather low [37]. Interestingly, a concurrent study of the same ABCG2 transporter demonstrated that it could be successfully solubilized and purified in an active form in the presence of CHAPS [38]. CHAPS has also been used to purify the thromboxane A2 receptor, resulting in low yields of relatively active proteins [39]. Similarly, other experiments have demonstrated that addition of CHAPS is beneficial for the isolation of the active recombinant receptors 5HT1A [40], CB2 [41], and AA2A [42], as well as a number of other class A receptors that we investigated (unpublished data). However, no previous study with CHAPS has provided any information on the homogeneity and polydispersity of the solubilized or purified samples. Our present SEC and EM data on CHAPS-treated samples revealed heterogeneous particles of high molecular sizes, suggesting that CHAPS does not fully solubilize *P. pastoris* membranes but rather generates lipoprotein complexes of various dimensions that remain soluble after ultracentrifugation. It appears that such an environment is well suited for maintaining MT1 ligand-binding activity, whereas the monomeric receptors fully solubilized with Fos14 alone seemed to have lost this capacity. This detrimental role of Fos14 on the receptor activity thus raises questions about its mode of action at the molecular level. Fos14 is a lipid-like zwitterionic molecule that is known to be very efficient at destabilizing lipid-lipid and lipid-protein interactions. It is however unlikely that the lost of activity seen for MT1 may be due to a detergent-dependent unfolding of the receptor since similar studies conducted on other GPCRs have demonstrated the conservation of their secondary structure when purified in presence of Fos14 [43], [44]. A more plausible explanation would involve a Fos14-dependent dissociation of important lipids that may play a crucial role in the receptor ligand binding activity. Alternatively, considering the length of the alkyl chain of Fos14, another possibility detailed in a recent report [45] would be related to the size and shape of the Fos14 micelle surrounding MT1 that would hinder the melatonin-binding site and thus interfere with the binding assay. Further investigations are therefore needed to understand the actual effect of Fos14 on MT1 activity and would be very helpful to evaluate the potential interest of this detergent for the study of other GPCRs.

Meanwhile, we demonstrated in the present study that an optimal combination of Fos14 and CHAPS was able to minimize the negative behavior of both detergents, allowing us to recover a relatively pure, homogeneous, monomeric, and ligand-binding competent MT1 receptor suitable for use in *in vitro* studies. Whether the performance of this detergent mixture is specific to MT1 isolated from *P. pastoris* membranes or may be successfully applied to other receptors and other organisms still need to be investigated. Because we already observed similar behaviors of CHAPS and Fos14 alone towards several other GPCRs, we are tempted to speculate that this detergent mix approach may prove beneficial not only for MT1.

The MT1 purified material was then compared with reference membrane samples, to extensively evaluate its molecular pharmacology towards a collection of 24 compounds from our melatonin library. Remarkably, the inhibition parameters measured in ligand-binding competition experiments for the 24 molecules revealed that the pharmacology of the purified receptor was very close to that of MT1 in CHO membranes. These findings validated the quality of the purified material, indicating that it may be of particular interest for primary screening of MT1-binding molecules. Purified MT1 samples could be very helpful for limiting the false positive rate usually experienced with classical screenings, since no cellular or membranous artifacts would be present to interfere with the assay [46], [47]. Obviously, this assay format would be unable to indicate the G-protein coupling and signaling properties of the compounds, which are known to be important, particularly in the melatoninergic system [19]. However, the purified receptor in detergent solutions can be considered as a starting point for subsequent insertion in a membrane-mimicking environment that allows the coupling to purified G proteins to be evaluated *in vitro*. As a proof of concept, we assembled here the detergent-solubilized MT1R into lipid nanodiscs. In these discs, the MT1R is able to activate its cognate Gαi partner in an agonist-dependent manner. Interestingly, the MT1R in the disc maintains not only its ability to activate G proteins in an agonist-dependent manner but also its ligand-independent basal activity. Of importance also, the occurrence of a single basal rate exchange value for Gi activation consistent with what has been described for other purified receptors suggests the occurrence of an homogeneous population of receptors in a native-like conformation. Further detailed investigations are ongoing, but the present data support the usefulness of such material for *in vitro* studies of MT1.

Interestingly, the present study results also highlighted a marked difference in ligand affinities when the receptor was assayed in *P. pastoris* membranes compared to the CHO samples. This is likely due to differences in membrane lipid composition between yeast and mammalian cells [48]. In particular, the major sterol entity in yeasts is ergosterol, while in animal cells it is cholesterol, which is reportedly essential for the activity of a growing number of GPCRs [49]. Therefore, the lower pKi values observed in *P. pastoris* membranes could be related to the lack of cholesterol that is not compensated by the yeast ergosterol, similar to previous observations for the human mu-opioid receptor expressed in the yeast *Saccharomyces cerevisiae* [50]. The CHO-like pKi values restored in MT1 purified from the yeast membranes was consistently linked to the presence of the cholesterol derivative CHS that we added to the detergent solutions. Such cholesterol supplementation is systematically employed for the preparation of GPCRs for structural studies, and its effect on the stability of purified receptors is well documented [51]. Such interpretations suggest that the ligand binding properties of MT1 would be modulated by the presence of cholesterol. It remains to be investigated whether such modulation occurs, and whether it depends on a direct interaction with cholesterol, as described for B2AR [52], or is linked to a targeted localization of MT1 in cholesterol-enriched membrane microdomains.

In conclusion, the present study describes the first successful attempt to produce and purify a melatonin receptor in such a state that it recognizes multiple ligands with affinities similar to those measured on membranes from higher eukaryotic cells expressing this receptor. This purified material represents a resource of choice for a number of *in vitro* studies and applications, including primary ligand screening approaches and structure–function investigations. In the future, the global strategy described here should be useful for other GPCRs that have proven difficult to produce and purify for biochemical and biophysical analyses.

## Supporting Information

Figure S1 **Purification of MT1 in presence of CHAPS.** Left panels: original SDS-PAGE Coomassie blue stained (A) or revealed by anti-Flag western blot (C) for various elution fractions obtained after the anti-Flag (E1 and E2) and the SEC (16 to 24) purification steps. Right panels: lanes corresponding to SEC fractions of interest (F17 and F22) were extracted from the original SDS-PAGE pictures and were assembled to generate the Coomassie Blue (B) and anti-Flag western blot (D) pictures used in figure 3A.2.
(DOCX)

Figure S2 **Purification of MT1 in presence of Fos14.** Left panels: original SDS-PAGE Coomassie blue stained (A) or revealed by anti-Flag western blot (C) for various elution fractions obtained after the anti-Flag (E1 and E2) and the SEC (16 to 24) purification steps. Right panels: lanes corresponding to SEC fractions of interest (F17 and F22) were extracted from the original SDS-PAGE pictures and were assembled to generate the Coomassie Blue (B) and anti-Flag western blot (D) pictures used in figure 3B.6.
(DOCX)

Figure S3 **Purification of MT1 in presence of Fos14 and CHAPS.** Left panels: original SDS-PAGE Coomassie blue stained (A) or revealed by anti-Flag western blot (C) obtained for various elution fractions of the SEC purification (17 to 24). Right panels: lanes corresponding to SEC fractions of interest (F17 and F22) were extracted from the original SDS-PAGE pictures and were assembled to generate the Coomassie Blue (B) and anti-Flag western blot (D) pictures used in figure 4.1.
(DOCX)
