# Supplementary material for: Recombinant Human Melatonin Receptor MT1 Isolated in Mixed Detergents Shows Pharmacology Similar to That in Mammalian Cell Membranes
Source: PLoS One. 2014 Jun 24;9(6):e100616. doi: 10.1371/journal.pone.0100616 (PMC4069108; doi:10.1371/journal.pone.0100616)

**FIGURE S2.** **Purification of MT1 in presence of Fos14.** Left panels: original SDS-PAGE Coomassie blue stained (A) or revealed by anti-Flag western blot (C) for various elution fractions obtained after the anti-Flag (E1 and E2) and the SEC (16 to 24) purification steps. Right panels: lanes corresponding to SEC fractions of interest (F17 and F22) were extracted from the original SDS-PAGE pictures and were assembled to generate the Coomassie Blue (B) and anti-Flag western blot (D) pictures used in figure 3B.6.


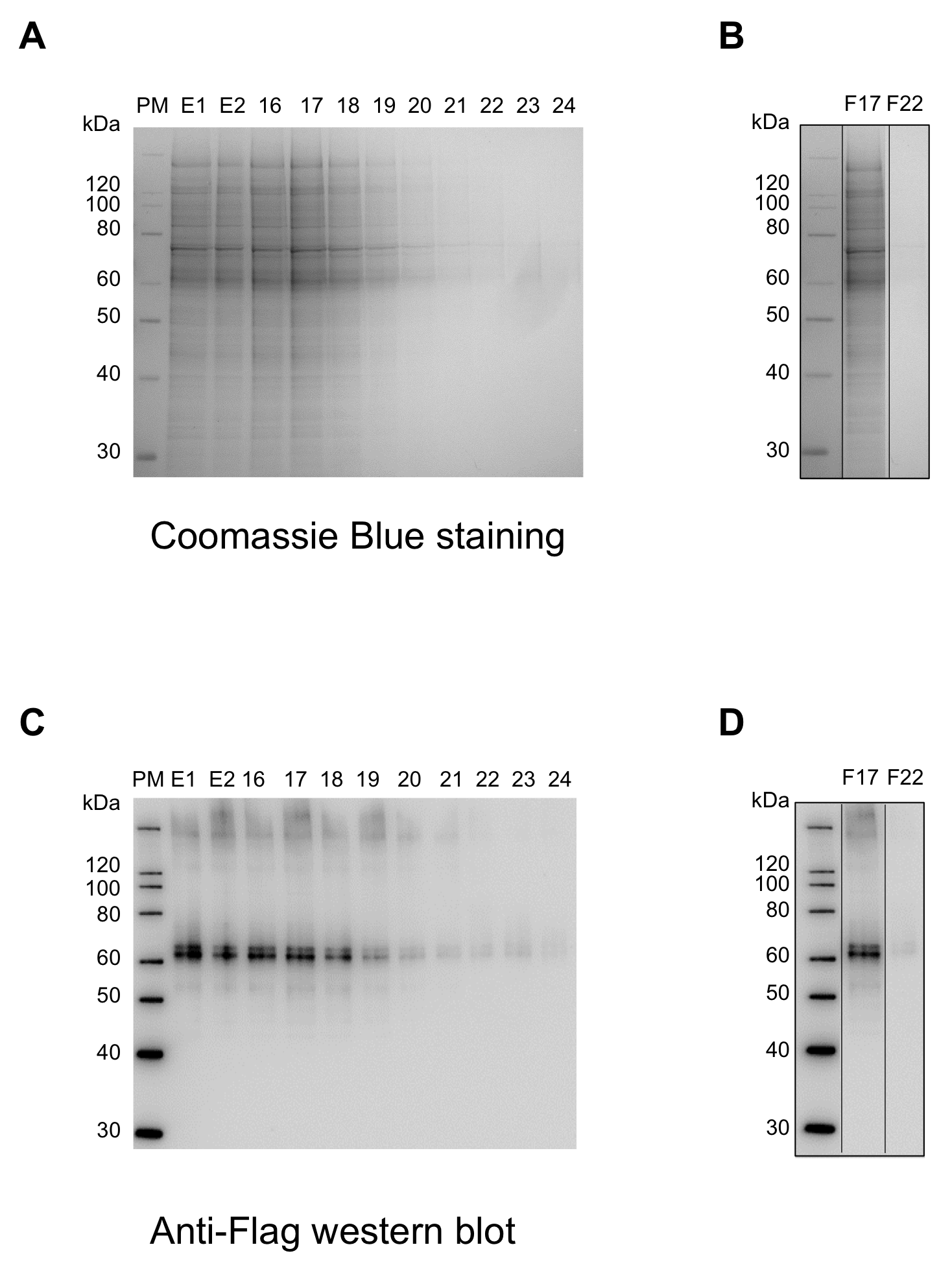

Supplement: Figure S2 — Purification of MT1 in presence of Fos14. Left panels: original SDS-PAGE Coomassie blue stained (A) or revealed by anti-Flag western blot (C) for various elution fractions obtained after the anti-Flag (E1 and E2) and the SEC (16 to 24) purification steps. Right panels: lanes corresponding to SEC fractions of interest (F17 and F22) were extracted from the original SDS-PAGE pictures and were assembled to generate the Coomassie Blue (B) and anti-Flag western blot (D) pictures used in figure 3B.6. (DOCX) [file pone.0100616.s002.docx]
