# Supplementary material for: Recombinant Human Melatonin Receptor MT1 Isolated in Mixed Detergents Shows Pharmacology Similar to That in Mammalian Cell Membranes
Source: PLoS One. 2014 Jun 24;9(6):e100616. doi: 10.1371/journal.pone.0100616 (PMC4069108; doi:10.1371/journal.pone.0100616)

**FIGURE S3.** **Purification of MT1 in presence of Fos14 and CHAPS.** Left panels: original SDS-PAGE Coomassie blue stained (A) or revealed by anti-Flag western blot (C) obtained for various elution fractions of the SEC purification (17 to 24). Right panels: lanes corresponding to SEC fractions of interest (F17 and F22) were extracted from the original SDS-PAGE pictures and were assembled to generate the Coomassie Blue (B) and anti-Flag western blot (D) pictures used in figure 4.1.


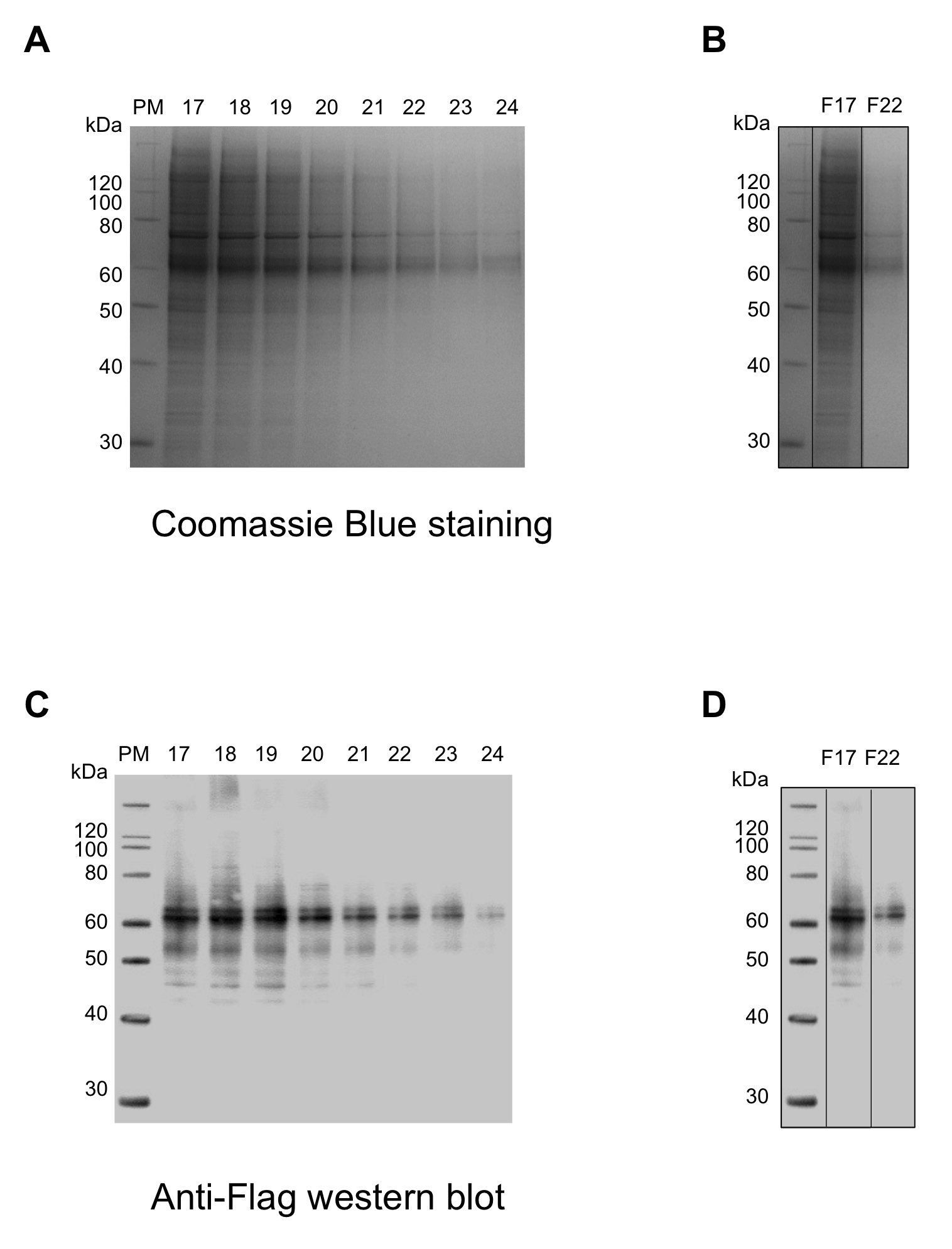

Supplement: Figure S3 — Purification of MT1 in presence of Fos14 and CHAPS. Left panels: original SDS-PAGE Coomassie blue stained (A) or revealed by anti-Flag western blot (C) obtained for various elution fractions of the SEC purification (17 to 24). Right panels: lanes corresponding to SEC fractions of interest (F17 and F22) were extracted from the original SDS-PAGE pictures and were assembled to generate the Coomassie Blue (B) and anti-Flag western blot (D) pictures used in figure 4.1. (DOCX) [file pone.0100616.s003.docx]
